# Production of Reactive Oxygen Species by Photosystem II as a Response to Light and Temperature Stress

**DOI:** 10.3389/fpls.2016.01950

**Published:** 2016-12-26

**Authors:** Pavel Pospíšil

**Affiliations:** Department of Biophysics, Centre of the Region Haná for Biotechnological and Agricultural Research, Faculty of Science, Palacký UniversityOlomouc, Czechia

**Keywords:** photoinhibition, heat inactivation, singlet oxygen, free oxygen radicals, lipid peroxidation

## Abstract

The effect of various abiotic stresses on photosynthetic apparatus is inevitably associated with formation of harmful reactive oxygen species (ROS). In this review, recent progress on ROS production by photosystem II (PSII) as a response to high light and high temperature is overviewed. Under high light, ROS production is unavoidably associated with energy transfer and electron transport in PSII. Singlet oxygen is produced by the energy transfer form triplet chlorophyll to molecular oxygen formed by the intersystem crossing from singlet chlorophyll in the PSII antennae complex or the recombination of the charge separated radical pair in the PSII reaction center. Apart to triplet chlorophyll, triplet carbonyl formed by lipid peroxidation transfers energy to molecular oxygen forming singlet oxygen. On the PSII electron acceptor side, electron leakage to molecular oxygen forms superoxide anion radical which dismutes to hydrogen peroxide which is reduced by the non-heme iron to hydroxyl radical. On the PSII electron donor side, incomplete water oxidation forms hydrogen peroxide which is reduced by manganese to hydroxyl radical. Under high temperature, dark production of singlet oxygen results from lipid peroxidation initiated by lipoxygenase, whereas incomplete water oxidation forms hydrogen peroxide which is reduced by manganese to hydroxyl radical. The understanding of molecular basis for ROS production by PSII provides new insight into how plants survive under adverse environmental conditions.

## Introduction

Photosystem II (PSII) is water-plastoquinone oxidoreductase embedded in the thylakoid membrane that catalyzes light-driven H_2_O oxidation to O_2_ and plastoquinone (PQ) reduction to plastoquinol (PQH_2_; Dau et al., 2012; Vinyard et al., 2013; Nelson and Junge, 2015; Suga et al., 2015; Najafpour et al., 2016). In this reaction, primary charge separation between the chlorophyll monomer (Chl_D1_) and pheophytin (Pheo_D1_) of D1 protein forms ^1^[Chl_D1_^•+^Pheo_D1_^•–^] radical pair which is fast stabilized by the oxidation of the weakly coupled chlorophyll dimer P_D1_ and P_D2_ (P680) forming ^1^[P680^•+^Pheo_D1_^•–^] radical pair (Cardona et al., 2012). ^1^[P680^•+^Pheo_D1_^•–^] radical pair is stabilized by the electron transport from Pheo_D1_ to the tightly bound plastoquinone Q_A_ forming Q_A_^•–^ and from the redox active tyrosine residue D1:161Y (Y_Z_) to P680^•+^ forming Y_Z_^•^. Electron transport form Q_A_^•–^ to loosely bound plastoquinone Q_B_ and the reduction of Y_Z_^•^ by the proton-coupled electron transport from the Mn_4_O_5_Ca cluster forms reducing and oxidizing equivalent at Q_B_ and Mn_4_O_5_Ca cluster, respectively. When two reducing equivalents are formed at Q_B_ site, its protonation forms plastoquinol (PQH_2_) which is liberated to PQ pool via channels (Lambreva et al., 2014). Formation of four oxidizing equivalents in the Mn_4_O_5_Ca cluster causes four-electron oxidation of two H_2_O to O_2_ which is released via channels into the lumen (Vogt et al., 2015).

Light-driven processes comprising both energy transfer and electron transport are accompanied by formation of reactive oxygen species (ROS). In the energy transfer, singlet oxygen (^1^O_2_) is formed by the energy transfer from triplet chlorophyll to O_2_ (Triantaphylides and Havaux, 2009; Pospíšil, 2012; Fischer et al., 2013). In electron transport, ROS are formed by the consecutive one-electron reduction of O_2_ and by the concerted two-electron oxidation of H_2_O on the PSII electron acceptor and donor sides, respectively (Pospíšil, 2009). The one-electron reduction of O_2_ forms superoxide anion radical (O_2_^•–^) which dismutes spontaneously or enzymatically to hydrogen peroxide (H_2_O_2_) and subsequently is reduced to hydroxyl radical (HO^•^) via Fenton reaction. The two-electron oxidation of water forms H_2_O_2_ which is oxidized and reduced to O_2_^•–^ and HO^•^, respectively. Non-enzymatic and enzymatic scavenging systems have been engaged to eliminate ROS and thus control level of ROS formed under various types of abiotic (adverse environmental conditions such as high light, high and low temperatures, UV-radiation, and drought) and biotic (herbivores and pathogens such as viruses, bacteria, and fungi) stresses.

Under moderate stress, when scavenging system maintains ROS level low, ROS serves as signaling molecules which activate an acclimation response and programmed cell death (Apel and Hirt, 2004; Dietz et al., 2016). Several lines of evidence have been provided that ROS play a crucial role in intracellular signaling from the chloroplast to the nucleus under high light (Gollan et al., 2015; Laloi and Havaux, 2015) and high temperature (Sun and Guo, 2016). However, due high reactivity of ROS toward proteins and lipids, ROS diffusion is limited. It seems to be unlikely that ROS might transmit signal from the chloroplast to the nucleus. It is considered that products of protein oxidation and lipid peroxidation might serve as signaling molecules (Fischer et al., 2012). As ROS formed by energy transfer (^1^O_2_) and electron transport (H_2_O_2_) are produced simultaneously, it seems to be likely that their action in signaling pathways interferes. It was demonstrated that H_2_O_2_ antagonizes the ^1^O_2_ signaling pathways in the *flu* Arabidopsis mutant (Laloi et al., 2007).

Under severe stress, when scavenging system is unable to sufficiently eliminate undesirable ROS formation, PSII proteins and lipids might be oxidized by ROS. Several lines of evidence were provided in the last three decades on the oxidative damage of PSII proteins by ROS under high light (Aro et al., 1993) and high temperature (Yamamoto et al., 2008). It is widely accepted that ^1^O_2_ is major ROS responsible for oxidative modification of PSII proteins. Contrary, H_2_O_2_ has low capability to oxidize PSII protein; however, when free or protein-bound metals are available, HO^•^ formed by Fenton reaction oxidizes nearby proteins. It has to be pointed that experimental evidence on PSII protein oxidation was obtained *in vitro* and thus it remains to be clarified whether oxidative modification of PSII proteins by ROS occurs *in vivo*. Apart to involvement of ROS in PSII protein damage, the inhibition of *de novo* protein synthesis by ROS was proposed under high light (Nishiyama et al., 2006) and high temperature (Allakhverdiev et al., 2008). Whereas PSII protein oxidation is widely described, limited evidence has been provided on lipid peroxidation near PSII. It was shown that ^1^O_2_ formed in PSII initiates lipid peroxidation in the thylakoid membrane (Triantaphylides et al., 2008).

In this review, an update on the latest findings on molecular mechanism of ROS formation at high light and high temperature is presented. In spite of the fact that molecular mechanism of ROS formation is substantially different at high light and high temperature, high light regularly combined with high temperature might bring about more serious impact on ROS formation.

## High Light

When light energy which is driving force for photosynthetic reactions exceeds the photosynthetic capacity, a light-induced decline in photochemical activity in PSII denoted as photoinhibition occurs. Limitations in the energy transfer and electron transport result in the generation of ROS. Limitation in energy transfer occurs, when the excess energy absorbed by chlorophyll in the PSII antennae complex is not fully utilized in the PSII reaction center by charge separation. Under these conditions, singlet chlorophyll might be converted to deleterious triplet chlorophyll. To prevent formation of triplet chlorophyll, quenching of singlet chlorophyll to heat is maintained directly by xanthophylls or indirectly by the rearrangement of Lhcb protein by PsbS (Ruban et al., 2012). However, when quenching of singlet chlorophyll is not sufficient, singlet chlorophyll is converted to triplet chlorophyll which transfers energy to O_2_ forming ^1^O_2_. Limitation in electron transport on the PSII electron acceptor side is accompanied by full reduction of PQ pool. As the Q_B_ site becomes unoccupied by PQ due to the full reduction of PQ pool, forward electron from Q_A_ to Q_B_ is blocked. Under these conditions, back electron transport from Q_A_^•–^ to Pheo and consequent recombination of Pheo^•–^ with P680^•+^ forms deleterious triplet chlorophyll which transfer to O_2_ forming ^1^O_2_. Under highly reducing conditions, double reduction and protonation of Q_A_ might result in the release of Q_A_H_2_ from its binding site. To prevent double reduction of Q_A_, electron from Q_A_^•–^ leaks to O_2_ forming O_2_^•–^. Superoxide anion radical is eliminated by its spontaneous and enzymatic dismutation to H_2_O_2_. In the interior of the thylakoid membrane, O_2_^•–^ is eliminated by the intrinsic SOD activity of cyt *b*_559_, whereas O_2_^•–^ which diffuse out the thylakoid membrane is eliminated by FeSOD attached to the stromal side of the thylakoid membrane at the vicinity of PSII. Limitation in electron transport on the PSII electron donor side is associated with incomplete H_2_O oxidation catalyzed by the Mn_4_O_5_Ca cluster. Incomplete H_2_O oxidation results in the formation of H_2_O_2_ which serves as precursor for HO^•^. Under conditions, when H_2_O_2_ is not properly eliminated by catalase, HO^•^ is formed by Fenton reactions catalyzed by iron and manganese on the PSII electron acceptor and donor sides, respectively.

### Singlet Oxygen

Singlet oxygen is formed by the triplet-triplet energy transfer from triplet chlorophyll or triple carbonyl to O_2_. Triplet-triplet energy transfer from triplet chlorophyll to O_2_ occurs in both the PSII antennae complex and the PSII reaction center. In the PSII antennae complex, triplet chlorophyll is formed by the photosensitization reaction, whereas in PSII reaction center triplet chlorophyll is formed by the charge recombination of triplet radical pair ^3^[P680^•+^Pheo^•–^]. Triplet-triplet energy transfer from triplet carbonyl to O_2_ proceeds during lipid peroxidation initiated by ROS formed by light. Whereas ^1^O_2_ formation by the energy transfer from triplet chlorophyll is well documented and represents the main source of ^1^O_2_ at high light, ^1^O_2_ formation by the energy transfer from triplet carbonyls is rarely evidenced and has marginal contribution to the overall ^1^O_2_ formation.

#### Triplet Chlorophyll

**FIGURE 1:**
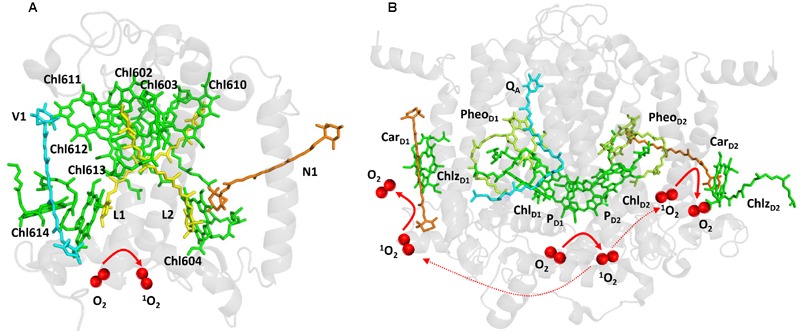
**Light-induced formation of ^1^O_2_ in the antennae complex (A)** and the reaction center **(B)** of PSII. The figures were made with Pymol (DeLano, 2002) using the structure for LHCII from *Spinacia oleracea* (PDB ID: 1rwt; Liu et al., 2004) and PSII from *Spinacia oleracea* (PDB ID: 3JCU; Wei et al., 2016).

Light energy absorbed by chlorophylls is transferred from the PSII antennae complex toward the PSII reaction center (van Amerongen and Croce, 2013). However, when energy transfer is limited, chlorophylls might serve as photosensitizers which form ^1^O_2_ by the energy transfer from their triplet state to O_2_ (**Figure 1A**). To prevent this, chlorophylls are coupled with carotenoids which have capability to quench triplet chlorophylls. Carotenoids consist of carotenes (β-carotene) and their oxygenated derivatives xanthophylls (lutein, zeaxanthin; Domonkos et al., 2013). In the PSII antennae complex, lutein and zeaxanthin play a crucial role in triplet chlorophyll quenching (Dall’Osto et al., 2006, 2012). Whereas lutein is permanently coordinated to Lhcb proteins, zeaxanthin is accumulated under high light by the reversible de-epoxidation of violaxanthin and is either free in the thylakoid membrane or bound to Lhcb protein (Havaux and Niyogi, 1999; Pinnola et al., 2013). Four xanthophyll binding sites were documented in the monomeric (Lhcb4-6) and the trimeric (LHCII) antenna proteins of PSII (Liu et al., 2004). Xanthophylls bound in both L1 (lutein) and L2 (lutein in LHCII and lutein or zeaxanthin in monomeric Lhcb4-6 proteins) sites can efficiently quench the neighboring triplet chlorophylls. Lutein in L1 (Lut620) and L2 (Lut621) are coupled with chlorophylls Chl610-Chl614 and Chl602- Chl604, respectively. The quenching of triplet chlorophylls 602 and 603 by lutein in L2 is highly efficient, whereas lutein in L1 site had no effect on quenching of triplet chlorophyll 612 (Ballottari et al., 2013). To maintain effective quenching of triplet chlorophyll by carotenoids, carotenoids has to be properly distanced and oriented from chlorophylls. Triplet-triplet energy transfer from chlorophylls to carotenoids is mediated by Dexter mechanism (Dexter, 1953), which needs overlap between the electron clouds of the donor and acceptor. When distance or orientation of carotenoid and chlorophyll is changed, the capability of carotenoids to quench excitation energy of triplet chlorophylls is diminished (Cupellini et al., 2016). Under such conditions, when O_2_ is in the proximity of triplet chlorophyll, the transfer of excitation energy from triplet chlorophyll to O_2_ forms ^1^O_2_. Comparison of the monomeric and the trimeric antenna proteins of PSII showed that the monomeric antenna proteins (Lhcb6 > Lhcb5 > Lhcb4) produced more ^1^O_2_ as compared to trimeric antenna proteins (LHCII; Ballottari et al., 2013).

When electron transport on the PSII electron acceptor side is limited due to the slow electron transport to the Q_A_ and Q_B_, several types of charge recombination of [P680^•+^ Q_A_^•–^] and ^1^[P680^•+^Pheo_D1_^•–^] radical pairs occur. Whereas [P680^•+^ Q_A_^•–^] radical pair recombines solely to the ground state P680, primary radical pair ^1^[P680^•+^Pheo_D1_^•–^] formed by the reverse electron transport from Q_A_^•–^ to Pheo_D1_ either recombines to the ground state P680 or converts to the triplet radical pair ^3^[P680^•+^Pheo_D1_^•–^] by change in the spin orientation. Recombination of triplet radical pair ^3^[P680^•+^Pheo_D1_^•–^] forms triplet chlorophyll ^3^P680^∗^ delocalized on the weakly coupled chlorophyll dimer P_D1_ and P_D2_ (Fischer et al., 2013; Telfer, 2014). Evidence has been provided that triplet state is localized on the Chl_D1_ at low temperature (Noguchi et al., 2001). The formation of ^3^Chl_D1_ was proposed to occur either directly by the charge recombination of the triplet radical pair ^3^[P680^•+^Pheo_D1_^•–^] or by the triplet energy transfer from ^3^P680^∗^ to Chl_D1_. As two β-carotenes (Car_D1_ and Car_D2_) are distanced from chlorophyll dimer P_D1_ and P_D2_, β-carotenes are not able to quench triplet chlorophyll ^3^P680^∗^ (**Figure 1B**).

#### Triplet Carbonyl

Lipid peroxidation initiated by radical ROS (O_2_^•–^, HO^•^) forms the primary and the secondary lipid peroxidation products. The primary lipid peroxidation product are lipid hydroperoxides (lipid hydroperoxy fatty acids, LOOH) which decompose to the secondary lipid peroxidation products lipid hydroxides (hydroxy fatty acids, LOH), reactive carbonyl species (RCS), and electronically excited species. Hydrogen abstraction from polyunsaturated fatty acid by HO^•^ forms lipid alkyl radical (L^•^) which interacts with O_2_ forming lipid peroxyl radical (LOO^•^). Lipid peroxyl radical abstracts hydrogen from the adjacent polyunsaturated fatty acid forming LOOH. Lipid hydroperoxide is stable; however, under oxidizing or reducing condition it is oxidized or reduced to LOO^•^ or alkoxyl radical (LO^•^). Cyclization or recombination of LOO^•^ forms high energy intermediates, dioxetane, or tetroxide. High energy intermediates are highly unstable and decomposite to triplet excited carbonyls (^3^L^∗^) which might transfer triplet energy to O_2_ forming ^1^O_2_. Alternatively, tetroxide might directly decompose to ^1^O_2_ via the Russell mechanism. Evidence has been provided that ^1^O_2_ is formed through lipid peroxidation under light stress in spinach PSII membranes deprived by the Mn_4_O_5_Ca cluster (Yadav and Pospíšil, 2012a). The authors demonstrated that the oxidation of lipids by highly oxidizing P680^•+^ and TyrZ^•^ caused ^1^O_2_ formation via the Russell mechanism. It has to be noted that amount of ^1^O_2_ formed by the triplet-triplet energy transfer from triplet chlorophyll is considerably higher than from triplet carbonyl.

### Superoxide Anion Radical

**FIGURE 2:**
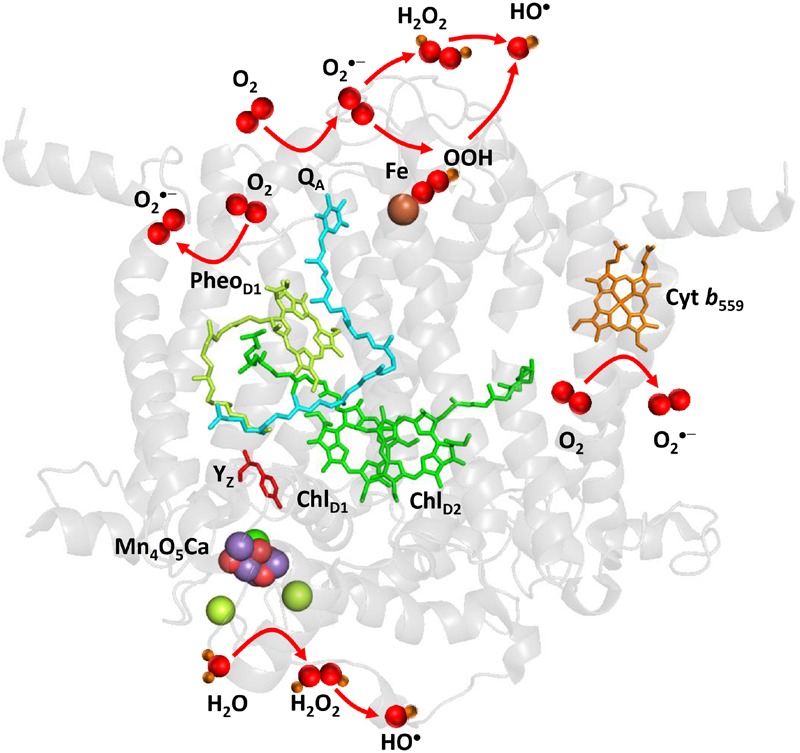
**Light-induced formation of O_2_^•–^, H_2_O_2_, and HO^•^ by PSII.** The figure was made with Pymol (DeLano, 2002) using the structure for PSII from *Spinacia oleracea* (PDB ID: 3JCU; Wei et al., 2016). Loosely bound plastoquinones (Q_B_ and Q_C_) were not resolved.

Superoxide anion radical is formed by the one-electron reduction of O_2_ on the PSII electron acceptor side (**Figure 2**). Pheophytin (Pheo_D1_^•–^), tightly bound plastosemiquinone (Q_A_^•–^), loosely bound plastosemiquinones (Q_B_^•–^ or Q_C_^•–^), free PQ (PQ^•–^), and ferrous iron of LP form of cyt *b*_559_ were proposed to serve as electron donors to O_2_ (Ananyev et al., 1994; Cleland and Grace, 1999; Pospíšil et al., 2004, 2006; Yadav et al., 2014). As Pheo_D1_^•–^ has highly negative redox potential, the reduction of O_2_ by Pheo_D1_^•–^ is thermodynamically feasible; however, its short lifetime makes the diffusion limited reduction of O_2_ less reasonable. Contrary, plastosemiquinones (Q_A_^•–^, Q_B_^•–^) does not fulfill thermodynamic criteria due to their more positive redox potential, whereas they accomplish the kinetic criteria due their long lifetime. However, due to the different concentration of O_2_ and O_2_^•–^, the standard redox potential of O_2_/O_2_^•–^ redox couple is shifted according Nernst equation to more positive and thus the reduction of O_2_ by plastosemiquinones becomes feasible (Pospíšil, 2009). The observation that exposure of isolated D1/D2/cyt *b*_559_ complexes which lacks Q_A_ to high light causes a significant rate of cytochrome (III) reduction revealed that Pheo_D1_^•–^ has capability to reduces O_2_. The detection of O_2_^•–^ in isolated thylakoids by a voltammetric method showed O_2_^•–^ production by the tightly bound plastosemiquinone Q_A_^•–^ (Cleland and Grace, 1999). Experimental evidence has been recently provided on the reduction of O_2_ by the loosely bound plastosemiquinones (Yadav et al., 2014). The authors demonstrated that plastosemiquinone is formed by the one-electron reduction of plastoquinone at the Q_B_ site and the one-electron oxidation of plastoquinol by cyt *b*_559_ at the Q_C_ site. Apart to cofactors involved in the linear transport, the ferrous heme iron of LP form of cyt *b*_559_ was shown to reduce O_2_ forming O_2_^•–^ (Pospíšil et al., 2006).

It has been demonstrated that PsbS knock-out rice mutants produced more O_2_^•–^ compared to WT under high light (Zulfugarov et al., 2014). The authors proposed that the lack of PsbS may cause shift in the midpoint redox potential of Q_A_/Q_A_^•–^ redox couple to more negative value and thus enhance O_2_^•–^ production by Q_A_^•–^. The D1 protein phosphorylation which is associated with the migration of damaged PSII complexes from the grana to the stroma lamellae during D1 protein repair cycle was shown to decrease O_2_^•–^ production (Chen et al., 2012). The author proposed that the D1 protein phosphorylation causes conformation change of D1 protein and thus modifies the binding of loosely bound plastosemiquinone to Q_B_ site. Consequently, the alternation of Q_B_ site brings about the decrease in O_2_^•–^ formed by the loosely bound plastosemiquinone Q_B_^•–^. In agreement with this proposal, it has been recently demonstrated that O_2_^•–^ production is enhanced in STN8 kinase knock-out rice mutants under high light (Poudyal et al., 2016). It has been proposed that enhancement in O_2_^•–^ production is due to the absence of conformational changes caused by STN8 kinase-induced phosphorylation. Using PsbY knock-out Arabidopsis plants, it has been shown that redox potential property of cyt *b*_559_ is controlled by PsbY protein (von Sydow et al., 2016). It has to be explored whether PsbY protein controls O_2_^•–^ production.

### Hydrogen Peroxide

**FIGURE 3:**
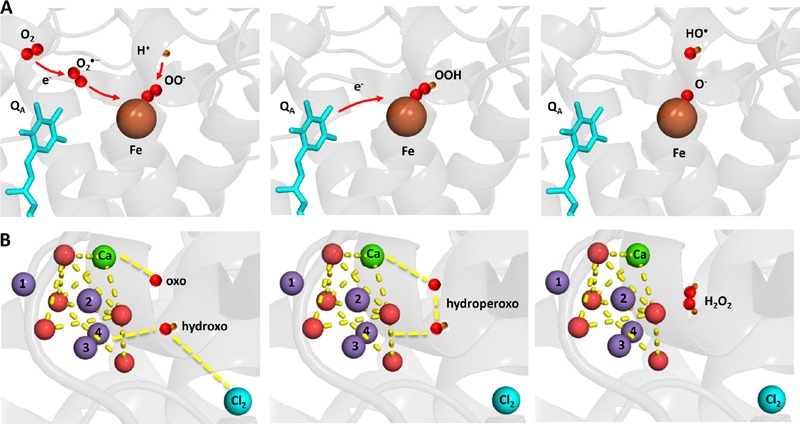
**Light-induced formation of bound peroxide and HO^•^ on the PSII electron acceptor (A)** donor **(B)** sides. The figure was made with Pymol (DeLano, 2002) using the structure for PSII from *Spinacia oleracea* (PDB ID: 3JCU; Wei et al., 2016).

Hydrogen peroxide is formed by the one-electron reduction of O_2_^•–^ and the two-electron oxidation of H_2_O on the PSII electron acceptor and donor sides, respectively (**Figure 2**). Hydrogen peroxide formation by the one-electron reduction of O_2_^•–^ occurs as dismutation or is maintained by plastosemiquinone. In the dismutation, two O_2_^•–^ are simultaneously reduced and oxidized forming H_2_O_2_ and O_2_, respectively. In the spontaneous dismutation, the interaction of two O_2_^•–^ is restricted due to repulsion of the negative charge on the molecule, whereas the interaction of the protonated form of superoxide, hydroperoxyl radical (HO_2_^•^), either with O_2_^•–^ or HO_2_^•^ is feasible. Spontaneous dismutation has been recently monitored by real-time detection of H_2_O_2_ in PSII membrane under high light using highly sensitive and selective osmium-horseradish modified electrode (Prasad et al., 2015). In the enzymatic dismutation, reduction and oxidation of O_2_^•–^ is associated with the redox change of the redox active metal center which serves as a superoxide oxidase (SOO) and superoxide reductase (SOR), respectively. It was demonstrated that the interaction of O_2_^•–^ with the non-heme iron results in the oxidation of the ferrous iron and the formation of ferric-peroxo species which is protonated to ferric-hydroperoxo species (bound peroxide; Pospíšil et al., 2004) (**Figure 3A**). Evidence has been provided that the ferric and ferrous heme irons of cyt *b*_559_ exhibit the SOO and the SOR activities, respectively (Tiwari and Pospíšil, 2009; Pospíšil, 2011). Apart to dismutation, free PQ^•–^ in PQ pool was proposed to participate in H_2_O_2_ formation. Hydrogen peroxide was shown to be formed by reduction of O_2_^•–^ by free PQ^•–^ (Borisova-Mubarakshina et al., 2015). The authors showed that H_2_O_2_ formed in PQ pool regulates the size of PSII antenna complex at high light. Furthermore, evidence has been provided that H_2_O_2_ might be formed by reduction ^1^O_2_ of by PQH_2_ (Khorobrykh et al., 2015). It was demonstrated that ^1^O_2_ generated by photosensitizer Rose Bengal interacts with PQH_2_ forming H_2_O_2_. The authors proposed that H_2_O_2_ formed by reduction of ^1^O_2_ by PQH_2_ in the thylakoid membrane might cause dimerization of the protein kinase STN7 and thus activates the enzyme.

Hydrogen peroxide formation by the two-electron oxidation of H_2_O is maintained by the Mn_4_O_5_Ca cluster when the complete four-electron oxidation of H_2_O to O_2_ is limited. Whereas all four manganese are redox active in four-electron oxidation of H_2_O to O_2_, the incomplete oxidation of H_2_O to H_2_O_2_ involves two redox active manganese. The two-electron oxidation of H_2_O has been proposed to involve the transition from either S_2_ to S_0_ state or S_1_ to S_-1_ state. Evidence has been provided that release of chloride from its binding site near to the Mn_4_O_5_Ca cluster enhanced H_2_O_2_ formation (Bradley et al., 1991; Fine and Frasch, 1992; Arato et al., 2004). A nucleophilic attack of hydroxo group on oxo group was proposed as an attractive model for formation of hydroperoxo species. It is proposed that nucleophilic attack of hydroxo group coordinated to Mn(4) and Cl(2) and oxo group coordinated to Ca forms hydroperoxo intermediate (**Figure 3B**). The hydroxo group is formed by deprotonation of the H_2_O substrate coordinated to to Mn(4) and Cl(2), whereas the oxo group is formed by double deprotonation of H_2_O substrate coordinated to Ca. A nucleophilic attack of manganese-coordinated hydroxo group on the calcium-coordinated electrophilic oxo group forms a peroxide intermediate that substitutes Cl(2) in coordination to Mn(4). Chloride controls accessibility of H_2_O substrate to Mn(4) and the nucleophilicity of hydroxo group and thus interaction of hydroxo and oxo groups. Water substrate, which serves as a precursor for the hydroxo group, enters into the catalytic site, when the Cl(2) binding site becomes opened to the solvent H_2_O due to its release.

### Hydroxyl Radical

Hydroxyl radical is formed by the one-electron reduction of H_2_O_2_ formed on the both PSII electron acceptor and donor sides (**Figure 2**). Hydroxyl radical formation by the one-electron reduction of free H_2_O_2_ and bound peroxide on the PSII electron acceptor side was shown to be maintained by free iron and the non-heme iron, respectively (Pospíšil et al., 2004). The authors demonstrated that the reduction of bound peroxide (ferric iron-hydroperoxo intermediate) formed by the interaction of O_2_^•–^ with the ferrous non-heme iron forms HO^•^ via ferric iron-oxo intermediate (**Figure 3A**).

Hydroxyl radical formation by the one-electron reduction of H_2_O_2_ on the PSII electron donor side is likely to be maintained by manganese. From thermodynamic point of view, the reduction of H_2_O_2_ by manganese is not feasible. It was proposed that the reduction of H_2_O_2_ by manganese becomes thermodynamically more favorable by (1) the coordination of manganese to the protein due to the decrease in the redox potential of manganese and (2) the pH decrease in the lumen due to the increase in the standard redox potential of H_2_O_2_/HO^•^ redox couple (Pospíšil, 2012). It was demonstrated that PSII membranes depleted by chloride shows higher HO^•^ formation compared to control PSII membranes (Arato et al., 2004). Based on the observation that HO^•^ formation was not completely suppressed by exogenous SOD, the authors proposed that HO^•^ is formed by reduction of H_2_O_2_ produced by the incomplete water oxidation on the PSII electron donor side.

## High Temperature

When PSII is exposed to high temperature, decline in the PSII activity denoted as heat inactivation occurs (Mathur et al., 2014). Heat inactivation occurs on the both PSII electron acceptor and donor sides. On the PSII electron donor side, heat inactivation is associated with the inhibition of water oxidation accompanied with release of PsbO, PsbP, and PsbQ proteins, calcium, chloride, and manganese from their binding sites (Coleman et al., 1988; Enami et al., 1994; Pospíšil et al., 2003; Barra et al., 2005). On the PSII electron acceptor side, heat inactivation is linked to the inhibition of electron transport from Q_A_ to Q_B_ (Pospíšil and Tyystjarvi, 1999). The authors demonstrated that increase in the midpoint redox potential of Q_A_/Q_A_^•–^ redox couple is responsible for the inhibition of Q_A_ to Q_B_ electron transport. Contrary to high light, ROS formation at high temperature is not driven by energy absorbed by chlorophylls; however, it is associated with heat-induced structural and functional changes in the thylakoid membrane. On the PSII electron acceptor side, ^1^O_2_ is formed decomposition of high energy intermediates formed by lipid peroxidation. On the PSII electron donor side, incomplete H_2_O oxidation forms H_2_O_2_ which is reduced by manganese to HO^•^ via Fenton reaction.

### Singlet Oxygen

Singlet oxygen is formed by the triplet-triplet energy transfer from ^3^L^∗^ to O_2_ produced by the decomposition of high energy intermediates, dioxetane, or tetroxide, formed during lipid peroxidation (Havaux et al., 2006; Pospíšil and Prasad, 2014). The observation that elimination of HO^•^ formation by mannitol did not suppress ^1^O_2_ formation revealed that lipid peroxidation is unlikely initiated by HO^•^ (Pospíšil et al., 2007). More recently, it has been demonstrated that inhibition of lipoxygenase by catechol and caffeic acid in *Chlamydomonas* cells prevented ^1^O_2_ formation (Prasad et al., 2016). Singlet oxygen was proposed to be generated at the lipid phase near the Q_B_ site (Yamashita et al., 2008). It was pointed that PQH_2_ formed by reduction of PQ by stromal reducing compound might cause ROS production which can damage D1 protein (Marutani et al., 2012).

### Hydrogen Peroxide

**FIGURE 4:**
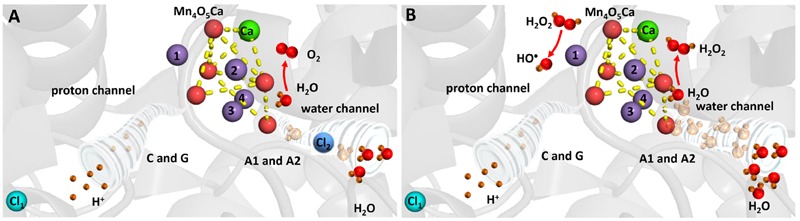
**Heat-induced formation of H_2_O_2_ and HO^•^ on the PSII electron donor side. (A)** Chloride controls accessibility of H_2_O to the Mn_4_O_5_Ca cluster and maintains complete oxidation of H_2_O to O_2_. **(B)** Removal of chloride results in uncontrolled accessibility of H_2_O to the Mn_4_O_5_Ca cluster and incomplete oxidation of H_2_O to H_2_O_2_. The figure was made with Pymol (DeLano, 2002) using the structure for PSII from *Spinacia oleracea* (PDB ID: 3JCU; Wei et al., 2016).

Hydrogen peroxide is formed by the two-electron oxidation of H_2_O on the PSII electron donor side (**Figure 4**). It was proposed that the release of extrinsic proteins (PsbO, PsbP, and PsbQ) leads to the inadequate accessibility of water to the Mn_4_O_5_Ca cluster and consequently to the formation of H_2_O_2_ (Thompson et al., 1989). Indeed, it was demonstrated using the amplex red fluorescent assay that exposure of PSII membranes to high temperature (40°C) results in H_2_O_2_ formation (Yadav and Pospíšil, 2012b). The authors demonstrated that the binding of acetate to the Mn_4_O_5_Ca cluster in the competition with chloride and blockage of water channel prevented H_2_O_2_ formation. Based on these observations, it was suggested that the release of chloride from its binding site near to the Mn_4_O_5_Ca cluster leads to uncontrolled accessibility of H_2_O to the Mn_4_O_5_Ca cluster. To maintain controlled four-electron oxidation of H_2_O to O_2_, the accessibility of H_2_O to the Mn_4_O_5_Ca cluster has to be regulated. Chloride coordinated to amino acids nearby the Mn_4_O_5_Ca cluster controls the accessibility of H_2_O to the metal center und thus maintain proper four-electron oxidation of H_2_O to O_2_. However, when chloride is released from its binding site, the delivery of H_2_O to the Mn_4_O_5_Ca cluster is unrestricted and incomplete oxidation of O_2_ to H_2_O_2_ occurs. Crystal structure of PSII from cyanobacteria *Thermosynechococcus vulcanus* reveals that two chlorides are located at distances of 6.67 and 7.40 Å from the Mn_4_O_5_Ca cluster (Umena et al., 2011). To avoid oxidation of nearby amino acid, diffusion of H_2_O_2_ into the lumen has to be restricted to the channels. As H_2_O_2_ is larger polar molecule similar to H_2_O, it seems to be likely that H_2_O_2_ diffuse into the lumen via water channels. However, when H_2_O_2_ leaks from the water channels, it might interact with manganese and formed HO^•^.

### Hydroxyl Radical

Hydroxyl radical is formed by the one-electron reduction of H_2_O_2_ formed on the PSII electron donor side (**Figure 4**). It was demonstrated by the EPR spin trapping spectroscopy that the exposure of PSII membranes to high temperature results in HO^•^ formation (Pospíšil et al., 2007). The authors showed that HO^•^ production is completely suppressed by exogenous catalase and metal chelator desferal revealing that HO^•^ is formed via the metal-catalyzed Fenton reaction. Furthermore, the observation that the addition of exogenous calcium and chloride prevented HO^•^ formation reveals that HO^•^ is produced by the Mn_4_O_5_Ca cluster. This proposal was confirmed by the observation that no HO^•^ formation was observed in PSII membranes deprived by the Mn_4_O_5_Ca cluster (Yamashita et al., 2008). As the replacement of chloride by acetate at its binding site near to the Mn_4_O_5_Ca cluster and the blockage of water channel prevented HO^•^ formation in a similar manner as H_2_O_2_ formation, it was assumed that chloride plays a crucial role in HO^•^ formation (Yadav and Pospíšil, 2012b). The authors proposed that H_2_O_2_ formed by the incomplete H_2_O oxidation is reduced to HO^•^ via the Fenton reaction mediated by free manganese released from the Mn_4_O_5_Ca cluster. The release of manganese from its binding site at high temperature was reported using atomic absorption (Nash et al., 1985) and EPR (Coleman et al., 1988; Pospíšil et al., 2003) spectroscopy. Detailed study using X-ray absorption spectroscopy showed that decomposition of the Mn_4_O_5_Ca cluster occurs in two steps (Pospíšil et al., 2003). In the first step, two manganese are released from their binding sites into the lumen remaining two manganese connected by a di-μ-oxo bridge, whereas in the second phase the remaining two manganese are liberated form PSII.

## Physiological Relevance of ROS Formation

### Role of ROS in Retrograde Signaling

Both ^1^O_2_ and H_2_O_2_ formed in the thylakoid membrane were proposed to be involved in retrograde signaling (Dietz et al., 2016). Role of ^1^O_2_ in acclimation and programmed cell death was demonstrated in green algae (Erickson et al., 2015) and higher plants (Triantaphylides and Havaux, 2009; Laloi and Havaux, 2015). In higher plants, *fluorescent* (*flu*) and *chlorina 1* (*ch1*) Arabidopsis mutants were advantageously used due to their high capability to form ^1^O_2_. It was proposed that the ^1^O_2_ level determines whether acclimation response or programmed cell death is triggered (Laloi and Havaux, 2015).

At low ^1^O_2_ level, acclimation response is mediated by β-cyclocitral formed by oxidation of β-carotene (Ramel et al., 2013a; Havaux, 2014). It was demonstrated that exposure of WT Arabidopsis plants to β-cyclocitral caused expression of ^1^O_2_ related gene (Ramel et al., 2012). In agreement with this finding, it was shown that concentration of β-cyclocitral is enhanced in *ch1* Arabidopsis plants under acclimation (Ramel et al., 2013b). Further, evidence was provided on the role of jasmonic acid in acclimation response. It was demonstrated that jasmonate-deficient Arabidopsis mutant (*delayed-dehiscence 2*) was more resistant to light and jasmonate biosynthesis was pronouncedly lowered under acclimation (Ramel et al., 2013b). Based on these observations, the authors proposed that downregulation of jasmonate biosynthesis plays a crucial role in the triggering of acclimation response (Ramel et al., 2013c).

At high ^1^O_2_ level, programmed cell death is dependent on the plastid proteins EXECUTER1 (EX1) and EXECUTER2 (EX2; Lee et al., 2007) and OXIDATIVE SIGNAL INDUCIBLE1 (OXI1) encoding an AGC kinase (Shumbe et al., 2016). Several lines of evidence on the involvement of EX1 and EX2 in programmed cell death were provided using *flu* Arabidopsis mutant (Lee et al., 2007). In this mutant, ^1^O_2_ is formed by triplet-triplet energy transfer from the triplet chlorophyll precursor protochlorophyllide to O_2_ (op den Camp et al., 2003). Even if EX1 and EX2 are located in chloroplast, it was proposed that jasmonic acid formed by ^1^O_2_-initiated lipid peroxidation mediates genetically controlled programmed cell death response via these two plastid proteins (Przybyla et al., 2008). The initiation of ^1^O_2_ signaling has been recently demonstrated close to EX1 in the grana margins nearby the site of chlorophyll synthesis and ^1^O_2_ formation (Wang et al., 2016). As ^1^O_2_ signaling depends on the FstH protease, the authors proposed that ^1^O_2_ signaling is linked to D1 repair cycle. Apart to EX1 and EX2, it has been shown recently that *OXI1* kinase is involved in ^1^O_2_ signaling in *ch1* Arabidopsis mutant (Shumbe et al., 2016). In this mutant, ^1^O_2_ is formed by triplet-triplet energy transfer from the triplet chlorophyll formed in PSII to O_2_ (Krieger-Liszkay, 2005). As *OXI1* kinase is localized at the cytosol at the cell periphery or in the nucleus, it seems to be likely that oxylipins mediate signal transduction from chloroplast to cytosol (Shumbe et al., 2016).

Hydrogen peroxide formed under high light was demonstrated to play a crucial role in signaling associated with acclimation and programmed cell death (Foyer and Noctor, 2009; Karpinski et al., 2013; Gollan et al., 2015). It is well established that H_2_O_2_ regulates expression of genes by the activation of protein kinase signaling pathways. It was proposed that precursor of jasmonic acid, 12-oxo phytodienoic acid (OPDA), mediates signal transduction from chloroplast to cytosol (Tikkanen et al., 2014). It has been recently demonstrated that H_2_O_2_ formed in PQ pool triggers signal transduction from the chloroplast to the nucleus via protein kinase signaling pathways leading to the regulation of the PSII antenna size during the acclimation response (Borisova-Mubarakshina et al., 2015).

Our knowledge on the involvement of ROS in retrograde signaling at high temperature is highly limited. While the physiological relevance of light-induced ^1^O_2_ to acclimation and programmed cell death is described to some extent, no evidence was provided on the role of ^1^O_2_ formed under high temperature to plant stress response. However, it seems to be likely that ^1^O_2_ might oxidize lipid, protein or pigment forming specific oxidation products and thus initiates signal transduction from the chloroplast to the nucleus in the signaling cascade pathway. Contrary to ^1^O_2_, H_2_O_2_ was shown to be an important component in heat stress-activated gene expression. Hydrogen peroxide was demonstrated to be involved in the synthesis of heat shock proteins (Volkov et al., 2006). More experimental data are required to pronouncedly progress our understanding of multiple signaling pathways involved the in response to heat stress.

### Role of ROS in Oxidative Damage

At high light, proteins and lipids might be oxidized by ROS formed in PSII. PSII proteins were evidenced to be oxidatively modified in the following order D1 > D2 > Cyt b559 > CP43 > CP47 > Mn_4_O_5_Ca cluster (Komenda et al., 2006). Amino acid oxidation at the lumen exposed AB-loop of D1 protein forms 24 kDa C-terminal and 9 kDa N-terminal fragments, whereas amino acid oxidation in the stromally exposed D-de loop of the D1 protein form 23-kDa N-terminal and 9-kDa C-terminal fragments (Edelman and Mattoo, 2008). Identification of naturally oxidized amino acid in D1 protein using mass spectrometry was shown nearby to the site of ROS production (Sharma et al., 1997; Frankel et al., 2012, 2013). Whereas D1 protein oxidation was pronouncedly studied *in vitro*, limited evidence was provided on D1 protein oxidation *in vivo* (Shipton and Barber, 1994; Lupinkova and Komenda, 2004). Regardless of a broad range of evidence on PSII protein oxidation obtained *in vitro*, the plausibility of these processes *in vivo* has to be clarify. An efficient repair cycle for D1 protein, which includes proteolytic degradation of damaged D1 protein and its replacement with a newly synthetized D1 copy is essential for maintaining the viability of PSII (Komenda et al., 2012; Mulo et al., 2012; Jarvi et al., 2015). Apart to involvement of ROS in PSII protein damage under high light, ROS were shown to suppress the synthesis *de novo* of proteins with the elongation step of translation as primary target (Nishiyama et al., 2006). However, considering the limited ROS diffusion, it seems to be more likely that ROS produced in the stroma might oxidize the translational elongation factors involved in D1 repair cycle. Unbound chlorophylls released to the stroma from their binding sites during PSII protein damage or chlorophyll precursors during chlorophyll synthesis are likely candidates for ^1^O_2_ formation due to the lack of effective quenching of triplet excitation energy by carotenoids. To avoid ^1^O_2_ formation, unbound chlorophylls might be temporarily coordinated to early light-induced proteins (ELIPs). In agreement with this proposal, it was demonstrated that small CAB-like proteins prevent ^1^O_2_ formation during PSII damage, most probably by the binding of unbound chlorophylls released from the damaged PSII complexes (Sinha et al., 2012). Lipids associated with membrane proteins were shown to be oxidized by ROS. The initiation of lipid peroxidation by ^1^O_2_ comprises the insertion of ^1^O_2_ to double bond of polyunsaturated fatty acid, whereas HO^•^ initiates lipid peroxidation by hydrogen abstraction from polyunsaturated fatty acid. It has been demonstrated that primary (LOOH) and secondary (LOH, RCS, and electronically excited species) lipid peroxidation products are formed at high light. Formation of hydroxy fatty acid was demonstrated in Arabidopsis plants (Triantaphylides et al., 2008). The authors showed that oxidation of polyunsaturated fatty acid by ^1^O_2_ leads to formation of LOOH which further forms LOH isomers (10-HOTE and 15-HOTE).

At high temperature, limited evidence was provided on the oxidation of proteins and lipids by ROS. It was demonstrated that exposure of thylakoid membranes to high temperature caused cleavage of D1 protein forming 9 kDa C-terminal and 23 kDa N-terminal fragments (Yoshioka et al., 2006). The authors demonstrated that FtsH protease is involved in the cleavage of the D1 protein at high temperature. Furthermore, it was reported that ^1^O_2_ formed at Q_B_ site by the recombination of LOO^•^ formed by the lipid peroxidation caused the D1 protein degradation by the interaction with D-*de* loop of the D1 protein in a similar manner as under high light (Yamashita et al., 2008). As experimental evidence for oxidative damage of PSII protein by endogenous ROS was obtained predominantly *in vitro*, it is unclear whether the PSII protein oxidation at high temperature occurs *in vivo*. Apart to involvement of ROS in PSII protein oxidation, the inhibition of *de novo* protein synthesis by ROS was proposed at high temperature (Allakhverdiev et al., 2008). Lipid peroxidation is associated with formation of RCS. It was demonstrated that malondialdehyde is formed in Arabidopsis plants exposed to heat stress (Yamauchi et al., 2008).

## Conclusion and Perspectives

Under environmental conditions, abiotic stresses adversely affect plant growth and survival. The impact of high light on the photosynthetic apparatus is considered to be of particular significance as light reactions of photosynthesis are inhibited prior to other cell functions are impaired. However, under environmental conditions, plants are exposed to combination of multiple stresses. High light stress is often associated with high temperature causing global warming which is one of the most important characteristics of accelerated climatic changes. Extensive research over the last 10 years focused on the structural and functional changes of the photosynthetic complexes in response to high light, high temperature or their combination. The exploration of molecular mechanism of ROS production by PSII helps to understand the adaptive processes by which plants cope with high light and high temperature stresses.

## Author Contributions

The PP wrote and approved manuscript for publication.

## Conflict of Interest Statement

The author declares that the research was conducted in the absence of any commercial or financial relationships that could be construed as a potential conflict of interest.

## References

[B1] Allakhverdiev S. I. Kreslavski V. D. Klimov V. V. Los D. A. Carpentier R. Mohanty P. (2008). Heat stress: an overview of molecular responses in photosynthesis. *Photosynth. Res.* 98 541–550. 10.1007/s11120-008-9331-0 18649006

[B2] Ananyev G. Renger G. Wacker U. Klimov V. (1994). The photoproduction of superoxide radicals and the superoxide-dismutase activity of photosystem-II - the possible involvement of cytochrome B559. *Photosynth. Res.* 41 327–338. 10.1007/BF00019410 24310115

[B3] Apel K. Hirt H. (2004). Reactive oxygen species: metabolism, oxidative stress, and signal transduction. *Annu. Rev. Plant Biol.* 55 373–399. 10.1146/annurev.arplant.55.031903.141701 15377225

[B4] Arato A. Bondarava N. Krieger-Liszkay A. (2004). Production of reactive oxygen species in chloride- and calcium-depleted photosystem II and their involvement in photoinhibition. *Biochim. Biophys. Acta* 1608 171–180. 10.1016/j.bbabio.2003.12.003 14871495

[B5] Aro E. M. Virgin I. Andersson B. (1993). Photoinhibition of Photosystem II. Inactivation, protein damage and turnover. *Biochim. Biophys. Acta* 1143 113–134. 10.1016/0005-2728(93)90134-2 8318516

[B6] Ballottari M. Mozzo M. Girardon J. Hienerwadel R. Bassi R. (2013). Chlorophyll triplet quenching and photoprotection in the higher plant monomeric antenna protein Lhcb5. *J. Phys. Chem. B* 117 11337–11348. 10.1021/jp402977y 23786371

[B7] Barra M. Haumann M. Dau H. (2005). Specific loss of the extrinsic 18 Kda protein from Photosystem II upon heating to 47 degrees C causes inactivation of oxygen evolution likely due to Ca release from the Mn-complex. *Photosynth. Res.* 84 231–237. 10.1007/s11120-004-7158-x 16049779

[B8] Borisova-Mubarakshina M. M. Ivanov B. N. Vetoshkina D. V. Lubimov V. Y. Fedorchuk T. P. Naydov I. A. (2015). Long-term acclimatory response to excess excitation energy: evidence for a role of hydrogen peroxide in the regulation of photosystem II antenna size. *J. Exp. Bot.* 66 7151–7164. 10.1093/jxb/erv410 26324464

[B9] Bradley R. L. Long K. M. Frasch W. D. (1991). The involvement of photosystem-ii-generated H2o2 in photoinhibition. *FEBS Lett.* 286 209–213. 10.1016/0014-5793(91)80975-9 1864370

[B10] Cardona T. Sedoud A. Cox N. Rutherford A. W. (2012). Charge separation in Photosystem II: a comparative and evolutionary overview. *Biochim. Biophys. Acta* 1817 26–43. 10.1016/j.bbabio.2011.07.012 21835158

[B11] Chen L. B. Jia H. Y. Tian Q. Du L. B. Gao Y. L. Miao X. X. (2012). Protecting effect of phosphorylation on oxidative damage of D1 protein by down-regulating the production of superoxide anion in photosystem II membranes under high light. *Photosynth. Res.* 112 141–148. 10.1007/s11120-012-9750-9 22644478

[B12] Cleland R. E. Grace S. C. (1999). Voltammetric detection of superoxide production by photosystem II. *FEBS Lett.* 457 348–352. 10.1016/S0014-5793(99)01067-4 10471806

[B13] Coleman W. J. Govindjee Gutowsky H. S. (1988). The effect of chloride on the thermal inactivation of oxygen evolution. *Photosynth. Res.* 16 261–276. 10.1007/BF00028844 24429532

[B14] Cupellini L. Jurinovich S. Prandi I. G. Caprasecca S. Mennucci B. (2016). Photoprotection and triplet energy transfer in higher plants: the role of electronic and nuclear fluctuations. *Phys. Chem. Chem. Phys.* 18 11288–11296. 10.1039/C6CP01437B 27052105

[B15] Dall’Osto L. Holt N. E. Kaligotla S. Fuciman M. Cazzaniga S. Carbonera D. (2012). Zeaxanthin protects plant photosynthesis by modulating chlorophyll triplet yield in specific light-harvesting antenna subunits. *J. Biol. Chem.* 287 41820–41834. 10.1074/jbc.M112.405498 23066020 PMC3516730

[B16] Dall’Osto L. Lico C. Alric J. Giuliano G. Havaux M. Bassi R. (2006). Lutein is needed for efficient chlorophyll triplet quenching in the major LHCII antenna complex of higher plants and effective photoprotection in vivo under strong light. *BMC Plant Biol.* 6:32 10.1186/1471-2229-6-32 PMC1769499 17192177

[B17] Dau H. Zaharieva I. Haumann M. (2012). Recent developments in research on water oxidation by photosystem II. *Curr. Opin. Chem. Biol.* 16 3–10. 10.1016/j.cbpa.2012.02.011 22387134

[B18] DeLano W. L. (2002). *The PYMOL Molecular Graphics System. Software.* Available at: http://www.pymol.org

[B19] Dexter D. L. (1953). A theory of sensitized luminescence in solids. *J. Chem. Phys.* 21 836–850. 10.1063/1.1699044

[B20] Dietz K. J. Turkan I. Krieger-Liszkay A. (2016). Redox- and reactive oxygen species-dependent signaling into and out of the photosynthesizing chloroplast. *Plant Physiol.* 171 1541–1550. 10.1104/pp.16.00375 27255485 PMC4936569

[B21] Domonkos I. Kis M. Gombos Z. Ughy B. (2013). Carotenoids, versatile components of oxygenic photosynthesis. *Prog. Lipid Res.* 52 539–561. 10.1016/j.plipres.2013.07.001 23896007

[B22] Edelman M. Mattoo A. K. (2008). D1-protein dynamics in photosystem II: the lingering enigma. *Photosynth. Res.* 98 609–620. 10.1007/s11120-008-9342-x 18709440

[B23] Enami I. Kitamura M. Tomo T. Isokawa Y. Ohta H. Katoh S. (1994). Is the primary cause of thermal inactivation of oxygen evolution in spinach PS-ii membranes release of the extrinsic 33 kda protein or of MN. *Biochim. Biophys. Acta* 1186 52–58. 10.1016/0005-2728(94)90134-1

[B24] Erickson E. Wakao S. Niyogi K. K. (2015). Light stress and photoprotection in *Chlamydomonas reinhardtii*. *Plant J.* 82 449–465. 10.1111/tpj.12825 25758978

[B25] Fine P. L. Frasch W. D. (1992). The oxygen-evolving complex requires chloride to prevent hydrogen-peroxide formation. *Biochemistry* 31 12204–12210. 10.1021/bi00163a033 1457417

[B26] Fischer B. B. Hideg E. Krieger-Liszkay A. (2013). Production, detection, and signaling of singlet oxygen in photosynthetic organisms. *Antioxid. Redox Signal.* 18 2145–2162. 10.1089/ars.2012.5124 23320833

[B27] Fischer B. B. Ledford H. K. Wakao S. Huang S. G. Casero D. Pellegrini M. (2012). Singlet oxygen resistant 1 links reactive electrophile signaling to singlet oxygen acclimation in *Chlamydomonas reinhardtii*. *Proc. Natl. Acad. Sci. U.S.A.* 109 E1302–E1311. 10.1073/pnas.1116843109 22529359 PMC3356615

[B28] Foyer C. H. Noctor G. (2009). Redox regulation in photosynthetic organisms: signaling, acclimation, and practical implications. *Antioxid. Redox Signal.* 11 861–905. 10.1089/ars.2008.2177 19239350

[B29] Frankel L. K. Sallans L. Limbach P. A. Bricker T. M. (2012). Identification of oxidized amino acid residues in the vicinity of the Mn4CaO5 cluster of Photosystem II: implications for the identification of oxygen channels within the photosystem. *Biochemistry* 51 6371–6377. 10.1021/bi300650n 22827410 PMC3448023

[B30] Frankel L. K. Sallans L. Limbach P. A. Bricker T. M. (2013). Oxidized amino acid residues in the vicinity of Q(A) and Pheo(D1) of the photosystem II reaction center: putative generation sites of reducing-side reactive oxygen species. *PLoS ONE* 8:e58042 10.1371/journal.pone.0058042 PMC3585169 23469138

[B31] Gollan P. J. Tikkanen M. Aro E.-M. (2015). Photosynthetic light reactions: integral to chloroplast retrograde signalling. *Curr. Opin. Plant Biol.* 27 180–191. 10.1016/j.pbi.2015.07.006 26318477

[B32] Havaux M. (2014). Carotenoid oxidation products as stress signals in plants. *Plant J.* 79 597–606. 10.1111/tpj.12386 24267746

[B33] Havaux M. Niyogi K. K. (1999). The violaxanthin cycle protects plants from photooxidative damage by more than one mechanism. *Proc. Natl. Acad. Sci. U.S.A.* 96 8762–8767. 10.1073/pnas.96.15.8762 10411949 PMC17590

[B34] Havaux M. Triantaphylides C. Genty B. (2006). Autoluminescence imaging: a non-invasive tool for mapping oxidative stress. *Trends Plant Sci.* 11 480–484. 10.1016/j.tplants.2006.08.001 16956784

[B35] Jarvi S. Suorsa M. Aro E. M. (2015). Photosystem II repair in plant chloroplasts - regulation, assisting proteins and shared components with photosystem II biogenesis. *Biochim. Biophys. Acta* 1847 900–909. 10.1016/j.bbabio.2015.01.006 25615587

[B36] Karpinski S. Szechynska-Hebda M. Wituszynska W. Burdiak P. (2013). Light acclimation, retrograde signalling, cell death and immune defences in plants. *Plant Cell Environ.* 36 736–744. 10.1111/pce.12018 23046215

[B37] Khorobrykh S. A. Karonen M. Tyystjarvi E. (2015). Experimental evidence suggesting that H2O2 is produced within the thylakoid membrane in a reaction between plastoquinol and singlet oxygen. *FEBS Lett.* 589 779–786. 10.1016/j.febslet.2015.02.011 25701589

[B38] Komenda J. Kuvikovi S. Lupinkova L. Masojidek J. (2006). “Biogenesis and structural dynamics of the photosystem II complex,” in *Biotechnological Applications of Photosynthetic Proteins: Biochips, Biosensors, and Biodevices* eds Giardi M. T. Piletska E. V. (New York, NY: Springer).

[B39] Komenda J. Sobotka R. Nixon P. J. (2012). Assembling and maintaining the photosystem II complex in chloroplasts and cyanobacteria. *Curr. Opin. Plant Biol.* 15 245–251. 10.1016/j.pbi.2012.01.017 22386092

[B40] Krieger-Liszkay A. (2005). Singlet oxygen production in photosynthesis. *J. Exp. Bot.* 56 337–346. 10.1093/jxb/erh237 15310815

[B41] Laloi C. Havaux M. (2015). Key players of singlet oxygen-induced cell death in plants. *Front. Plant Sci.* 6:39 10.3389/fpls.2015.00039 PMC4316694 25699067

[B42] Laloi C. Stachowiak M. Pers-Kamczyc E. Warzych E. Murgia I. Apel K. (2007). Cross-talk between singlet oxygen- and hydrogen peroxide-dependent signaling of stress responses in *Arabidopsis thaliana*. *Proc. Natl. Acad. Sci. U.S.A.* 104 672–677. 10.1073/pnas.0609063103 17197417 PMC1766442

[B43] Lambreva M. D. Russo D. Polticelli F. Scognamiglio V. Antonacci A. Zobnina V. (2014). Structure/function/dynamics of photosystem II plastoquinone binding sites. *Curr. Protein Pept. Sci.* 15 285–295. 10.2174/1389203715666140327104802 24678671 PMC4030317

[B44] Lee K. P. Kim C. Landgraf F. Apel K. (2007). EXECUTER1- and EXECUTER2-dependent transfer of stress-related signals from the plastid to the nucleus of *Arabidopsis thaliana*. *Proc. Natl. Acad. Sci. U.S.A.* 104 10270–10275. 10.1073/pnas.0702061104 17540731 PMC1891253

[B45] Liu Z. F. Yan H. C. Wang K. B. Kuang T. Y. Zhang J. P. Gui L. L. (2004). Crystal structure of spinach major light-harvesting complex at 2.*72* angstrom resolution. *Nature* 428 287–292. 10.1038/nature02373 15029188

[B46] Lupinkova L. Komenda J. (2004). Oxidative modifications of the Photosystem II D1 protein by reactive oxygen species: from isolated protein to cyanobacterial cells. *Photochem. Photobiol.* 79 152–162. 10.1562/0031-8655nyr(2004)079<0152:OMOTPI>2.0.CO;2 15068028

[B47] Marutani Y. Yamauchi Y. Kimura Y. Mizutani M. Sugimoto Y. (2012). Damage to photosystem II due to heat stress without light-driven electron flow: involvement of enhanced introduction of reducing power into thylakoid membranes. *Planta* 236 753–761. 10.1007/s00425-012-1647-5 22526503

[B48] Mathur S. Agrawal D. Jajoo A. (2014). Photosynthesis: response to high temperature stress. *J. Photochem. Photobiol. B Biol.* 137 116–126. 10.1016/j.jphotobiol.2014.01.010 24796250

[B49] Mulo P. Sakurai I. Aro E.-M. (2012). Strategies for psbA gene expression in cyanobacteria, green algae and higher plants: from transcription to PSII repair. *Biochim. Biophys. Acta* 1817 247–257. 10.1016/j.bbabio.2011.04.011 21565160

[B50] Najafpour M. M. Renger G. Hołyńska M. Moghaddam A. N. Aro E.-M. Carpentier R. (2016). Manganese compounds as water-oxidizing catalysts: from the natural water-oxidizing complex to nanosized manganese oxide structures. *Chem. Rev.* 116 2886–2936. 10.1021/acs.chemrev.5b00340 26812090

[B51] Nash D. Miyao M. Murata N. (1985). Heat inactivation of oxygen evolution in photosystem-II particles and its acceleration by chloride depletion and exogenous manganese. *Biochim. Biophys. Acta* 807 127–133. 10.1016/0005-2728(85)90115-X

[B52] Nelson N. Junge W. (2015). Structure and energy transfer in photosystems of oxygenic photosynthesis. *Annu. Rev. Biochem.* 84 659–683. 10.1146/annurev-biochem-092914-041942 25747397

[B53] Nishiyama Y. Allakhverdiev S. I. Murata N. (2006). A new paradigm for the action of reactive oxygen species in the photoinhibition of photosystem II. *Biochim. Biophys. Acta* 1757 742–749. 10.1016/j.bbabio.2006.05.013 16784721

[B54] Noguchi T. Tomo T. Kato C. (2001). Triplet formation on a monomeric chlorophyll in the photosystem II reaction center as studied by time-resolved infrared spectroscopy. *Biochemistry* 40 2176–2185. 10.1021/bi0019848 11329286

[B55] op den Camp R. G. L. Przybyla D. Ochsenbein C. Laloi C. Kim C. H. Danon A. (2003). Rapid induction of distinct stress responses after the release of singlet oxygen in arabidopsis. *Plant Cell* 15 2320–2332. 10.1105/tpc.014662 14508004 PMC197298

[B56] Pinnola A. Dall’osto L. Gerotto C. Morosinotto T. Bassi R. Alboresi A. (2013). Zeaxanthin binds to light-harvesting complex stress-related protein to enhance nonphotochemical quenching in *Physcomitrella patens*. *Plant Cell* 25 3519–3534. 10.1105/tpc.113.114538 24014548 PMC3809547

[B57] Pospíšil P. (2009). Production of reactive oxygen species by photosystem II. *Biochim. Biophys. Acta* 1787 1151–1160. 10.1016/j.bbabio.2009.05.005 19463778

[B58] Pospíšil P. (2011). Enzymatic function of cytochrome b(559) in photosystem II. *J. Photochem. Photobiol. B Biol.* 104 341–347. 10.1016/j.jphotobiol.2011.02.013 21377371

[B59] Pospíšil P. (2012). Molecular mechanisms of production and scavenging of reactive oxygen species by photosystem II. *Biochim. Biophys. Acta* 1817 218–231. 10.1016/j.bbabio.2011.05.017 21641332

[B60] Pospíšil P. Arato A. Krieger-Liszkay A. Rutherford A. W. (2004). Hydroxyl radical generation by Photosystem II. *Biochemistry* 43 6783–6792. 10.1021/bi036219i 15157112

[B61] Pospíšil P. Haumann M. Dittmer J. Sole V. A. Dau H. (2003). Stepwise transition of the tetra-manganese complex of photosystem II to a binuclear Mn-2(mu-O)(2) complex in response to a temperature jump: a time-resolved structural investigation employing X-ray absorption spectroscopy. *Biophys. J.* 84 1370–1386. 10.1016/S0006-3495(03)74952-2 12547817 PMC1302713

[B62] Pospíšil P. Prasad A. (2014). Formation of singlet oxygen and protection against its oxidative damage in Photosystem II under abiotic stress. *J. Photochem. Photobiol. B Biol.* 137 39–48. 10.1016/j.jphotobiol.2014.04.025 24928204

[B63] Pospíšil P. Snyrychova I. Kruk J. Strzalka K. Naus J. (2006). Evidence that cytochrome b(559) is involved in superoxide production in photosystem II: effect of synthetic short-chain plastoquinones in a cytochrome b(559) tobacco mutant. *Biochem. J.* 397 321–327. 10.1042/BJ20060068 16569212 PMC1513276

[B64] Pospíšil P. Šnyrychová I. Nauš J. (2007). Dark production of reactive oxygen species in photosystem II membrane particles at elevated temperature: EPR spin-trapping study. *Biochim. Biophys. Acta* 1767 854–859. 10.1016/j.bbabio.2007.02.011 17395149

[B65] Pospíšil P. Tyystjarvi E. (1999). Molecular mechanism of high-temperature-induced inhibition of acceptor side of Photosystem II. *Photosynth. Res.* 62 55–66. 10.1023/A:1006369009170

[B66] Poudyal R. S. Nath K. Zulfugarov I. S. Lee C. H. (2016). Production of superoxide from photosystem II-light harvesting complex II supercomplex in STN8 kinase knock-out rice mutants under photoinhibitory illumination. *J. Photochem. Photobiol. B* 162 240–247. 10.1016/j.jphotobiol.2016.06.050 27390892

[B67] Prasad A. Ferretti U. Sedlářová M. Pospíšil P. (2016). Singlet oxygen production in *Chlamydomonas reinhardtii* under heat stress. *Sci. Rep.* 6 20094 10.1038/srep20094 PMC4757480 26831215

[B68] Prasad A. Kumar A. Suzuki M. Kikuchi H. Sugai T. Kobayashi M. (2015). Detection of hydrogen peroxide in Photosystem II (PSII) using catalytic amperometric biosensor. *Front. Plant Sci.* 6:862 10.3389/fpls.2015.00862 PMC4606053 26528319

[B69] Przybyla D. Gobel C. Imboden A. Hamberg M. Feussner I. Apel K. (2008). Enzymatic, but not non-enzymatic, O-1(2)-mediated peroxidation of polyunsaturated fatty acids forms part of the EXECUTER1-dependent stress response program in the flu mutant of *Arabidopsis thaliana*. *Plant J.* 54 236–248. 10.1111/j.1365-313X.2008.03409.x 18182022

[B70] Ramel F. Birtic S. Ginies C. Soubigou-Taconnat L. Triantaphylides C. Havaux M. (2012). Carotenoid oxidation products are stress signals that mediate gene responses to singlet oxygen in plants. *Proc. Natl. Acad. Sci. U.S.A.* 109 5535–5540. 10.1073/pnas.1115982109 22431637 PMC3325660

[B71] Ramel F. Ksas B. Akkari E. Mialoundama A. S. Monnet F. Krieger-Liszkay A. (2013b). Light-induced acclimation of the *Arabidopsis* chlorina1 mutant to singlet oxygen. *Plant Cell* 25 1445–1462. 10.1105/tpc.113.109827 23590883 PMC3663279

[B72] Ramel F. Ksas B. Havaux M. (2013c). Jasmonate: a decision maker between cell death and acclimation in the response of plants to singlet oxygen. *Plant Signal. Behav.* 8 e26655 10.4161/psb.26655 PMC4091353 24103864

[B73] Ramel F. Mialoundama A. S. Havaux M. (2013a). Nonenzymic carotenoid oxidation and photooxidative stress signalling in plants. *J. Exp. Bot.* 64 799–805. 10.1093/jxb/ers223 22915744

[B74] Ruban A. V. Johnson M. P. Duffy C. D. P. (2012). The photoprotective molecular switch in the photosystem II antenna. *Biochim. Biophys. Acta* 1817 167–181. 10.1016/j.bbabio.2011.04.007 21569757

[B75] Sharma J. Panico M. Shipton C. A. Nilsson F. Morris H. R. Barber J. (1997). Primary structure characterization of the photosystem II D1 and D2 subunits. *J. Biol. Chem.* 272 33158–33166. 10.1074/jbc.272.52.33158 9407103

[B76] Shipton C. A. Barber J. (1994). In-vivo and in-vitro photoinhibition reactions generate similar degradation fragments of D1 and D2 photosystem-ii reaction-center proteins. *Eur. J. Biochem.* 220 801–808. 10.1111/j.1432-1033.1994.tb18682.x 8143734

[B77] Shumbe L. Chevalier A. Legeret B. Taconnat L. Monnet F. Havaux M. (2016). Singlet oxygen-induced cell death in *Arabidopsis* under high-light stress is controlled by OXI1 kinase. *Plant Physiol.* 170 1757–1771. 26747288 10.1104/pp.15.01546 PMC4775124

[B78] Sinha R. K. Komenda J. Knoppova J. Sedlářová M. Pospíšil P. (2012). Small CAB-like proteins prevent formation of singlet oxygen in the damaged photosystem II complex of the cyanobacterium *Synechocystis* sp PCC 6803. *Plant Cell Environ.* 35 806–818. 10.1111/j.1365-3040.2011.02454.x 22070528

[B79] Suga M. Akita F. Hirata K. Ueno G. Murakami H. Nakajima Y. (2015). Native structure of photosystem II at 1.95 angstrom resolution viewed by femtosecond X-ray pulses. *Nature* 517 99–103. 10.1038/nature13991 25470056

[B80] Sun A. Z. Guo F. Q. (2016). Chloroplast retrograde regulation of heat stress responses in plants. *Front. Plant Sci.* 7:398 10.3389/fpls.2016.00398 PMC4814484 27066042

[B81] Telfer A. (2014). Singlet oxygen production by PSII under light stress: mechanism, detection and the protective role of beta-carotene. *Plant Cell Physiol.* 55 1216–1223. 10.1093/pcp/pcu040 24566536 PMC4080269

[B82] Thompson L. K. Blaylock R. Sturtevant J. M. Brudvig G. W. (1989). Molecular-basis of the heat denaturation of photosystem-II. *Biochemistry* 28 6686–6695. 10.1021/bi00442a023 2675973

[B83] Tikkanen M. Gollan P. J. Mekala N. R. Isojarvi J. Aro E. M. (2014). Light-harvesting mutants show differential gene expression upon shift to high light as a consequence of photosynthetic redox and reactive oxygen species metabolism. *Philos. Trans. R. Soc. B Biol. Sci.* 369 20130229 10.1098/rstb.2013.0229 PMC3949394 24591716

[B84] Tiwari A. Pospíšil P. (2009). Superoxide oxidase and reductase activity of cytochrome b(559) in photosystem II. *Biochim. Biophys. Acta* 1787 985–994. 10.1016/j.bbabio.2009.03.017 19345666

[B85] Triantaphylides C. Havaux M. (2009). Singlet oxygen in plants: production, detoxification and signaling. *Trends Plant Sci.* 14 219–228. 10.1016/j.tplants.2009.01.008 19303348

[B86] Triantaphylides C. Krischke M. Hoeberichts F. A. Ksas B. Gresser G. Havaux M. (2008). Singlet oxygen is the major reactive oxygen species involved in photooxidative damage to plants. *Plant Physiol.* 148 960–968. 10.1104/pp.108.125690 18676660 PMC2556806

[B87] Umena Y. Kawakami K. Shen J. R. Kamiya N. (2011). Crystal structure of oxygen-evolving photosystem II at a resolution of 1.9 A. *Nature* 473 55–60. 10.1038/nature09913 21499260

[B88] van Amerongen H. Croce R. (2013). Light harvesting in photosystem II. *Photosynth. Res.* 116 251–263. 10.1007/s11120-013-9824-3 23595278 PMC3824292

[B89] Vinyard D. J. Ananyev G. M. Dismukes G. C. (2013). Photosystem II: the reaction center of oxygenic photosynthesis. *Annu. Rev. Biochem.* 82 577–606. 10.1146/annurev-biochem-070511-100425 23527694

[B90] Vogt L. Vinyard D. J. Khan S. Brudvig G. W. (2015). Oxygen-evolving complex of Photosystem II: an analysis of second-shell residues and hydrogen-bonding networks. *Curr. Opin. Chem. Biol.* 25 152–158. 10.1016/j.cbpa.2014.12.040 25621456

[B91] Volkov R. A. Panchuk I. I Mullineaux P. M. Schoﬄ F. (2006). Heat stress-induced H2O2 is required for effective expression of heat shock genes in *Arabidopsis*. *Plant Mol. Biol.* 61 733–746. 10.1007/s11103-006-0045-4 16897488

[B92] von Sydow L. Schwenkert S. Meurer J. Funk C. Mamedov F. Schröder W. P. (2016). The PsbY protein of *Arabidopsis* PhotosystemII is important for the redox control of cytochrome b559. *Biochim. Biophys. Acta* 1857 1524–1533. 10.1016/j.bbabio.2016.05.004 27220875

[B93] Wang L. S. Kim C. Xu X. Piskurewicz U. Dogra V. Singh S. (2016). Singlet oxygen- and EXECUTER1-mediated signaling is initiated in grana margins and depends on the protease FtsH2. *Proc. Natl. Acad. Sci. U.S.A.* 113 E3792–E3800. 10.1073/pnas.1603562113 27303039 PMC4932964

[B94] Wei X. Su X. Cao P. Liu X.-Y. Chang W. Li M. (2016). Structure of spinach photosystem II–LHCII supercomplex at 3.2 Å resolution. *Nature* 534 69–74. 10.1038/nature18020 27251276

[B95] Yadav D. K. Pospíšil P. (2012a). Evidence on the formation of singlet oxygen in the donor side photoinhibition of photosystem II: EPR spin-trapping study. *PLoS ONE* 7:e45883 10.1371/journal.pone.0045883 PMC3458798 23049883

[B96] Yadav D. K. Pospíšil P. (2012b). Role of chloride ion in hydroxyl radical production in photosystem II under heat stress: electron paramagnetic resonance spin-trapping study. *J. Bioenerg. Biomembr.* 44 365–372. 10.1007/s10863-012-9433-4 22466970

[B97] Yadav D. K. Prasad A. Kruk J. Pospíšil P. (2014). Evidence for the involvement of loosely bound plastosemiquinones in superoxide anion radical production in photosystem II. *PLoS ONE* 9:e115466 10.1371/journal.pone.0115466 PMC4277363 25541694

[B98] Yamamoto Y. Aminaka R. Yoshioka M. Khatoon M. Komayama K. Takenaka D. (2008). Quality control of photosystem II: impact of light and heat stresses. *Photosynth. Res.* 98 589–608. 10.1007/s11120-008-9372-4 18937045

[B99] Yamashita A. Nijo N. Pospíšil P. Morita N. Takenaka D. Aminaka R. (2008). Quality control of photosystem II - Reactive oxygen species are responsible for the damage to photosystem II under moderate heat stress. *J. Biol. Chem.* 283 28380–28391. 10.1074/jbc.M710465200 18664569 PMC2661399

[B100] Yamauchi Y. Furutera A. Seki K. Toyoda Y. Tanaka K. Sugimoto Y. (2008). Malondialdehyde generated from peroxidized linolenic acid causes protein modification in heat-stressed plants. *Plant Physiol. Biochem.* 46 786–793. 10.1016/j.plaphy.2008.04.018 18538576

[B101] Yoshioka M. Uchida S. Mori H. Komayama K. Ohira S. Morita N. (2006). Quality control of photosystem II. Cleavage of reaction center D1 protein in spinach thylakoids by FtsH protease under moderate heat stress. *J. Biol. Chem.* 281 21660–21669. 10.1074/jbc.M602896200 16735503

[B102] Zulfugarov I. S. Tovuu A. Eu Y.-J. Dogsom B. Poudyal R. S. Nath K. (2014). Production of superoxide from Photosystem II in a rice (*Oryza sativa* L.) mutant lacking PsbS. *BMC Plant Biol.* 14:242 10.1186/s12870-014-0242-2 PMC4219129 25342550

